# Efficacy and safety of super-mini percutaneous nephrolithotomy in the treatment of urinary calculi: a systematic review and meta-analysis

**DOI:** 10.1186/s12894-023-01256-z

**Published:** 2023-05-09

**Authors:** Han Li, Yong Yin, Ming Nie

**Affiliations:** 1 Urology of Chengdu First People’s Hospital, Chengdu Integrated Traditional Chinese Medicine and Western Medicine Hospital, Chengdu, Sichuan 610000 China; 2 Urology of Chengdu First People’s Hospital, Chengdu Integrated Traditional Chinese Medicine and Western Medicine Hospital, Chengdu City, Sichuan Province 610000 China

**Keywords:** Nephrolithiasis, Urolithiasis, Percutaneous nephrolithotomy, Super-mini-percutaneous nephrolithotomy, Complications, meta-analysis

## Abstract

**Background:**

Super-mini-percutaneous nephrolithotomy (SMP) is feasible and safe in adults and children with moderate-size renal calculi, but the use of SMP to remove larger calculi has yet to be determined. This study aimed to review the efficacy (stone-free rate, SFR) and safety of SMP in treating urinary calculi.

**Methods:**

PubMed, the Cochrane Library, and Embase were searched for eligible studies published up to May 2021. The primary outcome was the SFR. The secondary outcomes were the complications (using the Clavien-Dindo grading system), pain score, hospitalization days, and mean hemoglobin decline. All analyses were performed using the random-effects model. Nine studies (2433 patients with SMP and 2178 controls) were included.

**Results:**

SMP was not associated with an improved SFR in patients with calculi (RR = 1.05, 95%CI: 0.99–1.11). There were no differences in the occurrence of Clavien-Dindo I (RR = 0.95, 95%CI: 0.67–1.35) and Clavien-Dindo II (RR = 0.91, 95%CI: 0.58–1.42) complications between SMP and the control procedures. There were more Clavien-Dindo III complications with SMP than with the control procedures (RR = 0.71, 95%CI: 0.55–0.91), but none of the individual complications significantly differed between the two groups. Clavien-Dindo I fever appeared to be higher with SMP than with the control procedure (RR = 0.64, 95%CI: 0.50–0.83).

**Conclusion:**

In terms of efficacy, there were no differences between SMP and other procedures in treating urinary calculi. Clavien-Dindo I fever and Clavien-Dindo III complications might be more frequent with SMP than other procedures.

**Supplementary Information:**

The online version contains supplementary material available at 10.1186/s12894-023-01256-z.

## Background

Urolithiasis and nephrolithiasis are common health issues, with a global prevalence of 1.7-14.8% in 2010 and a rising incidence [1]. The prevalence of renal calculi in adults in China is 5.8% [2]. Although urolithiasis and nephrolithiasis can be asymptomatic for long periods, pain, infection, and obstruction can eventually occur [1, 3, 4]. The recurrence rate of urolithiasis and nephrolithiasis is high [3].

Percutaneous nephrolithotomy (PCNL) is currently considered standard for large or multiple renal calculi [1, 3–6]. Despite the high rate of calculi clearance using PCNL, there are possible complications, and the potentially most serious complication is bleeding [7]. Advanced equipment is being developed to prevent bleeding during PCNL [8], including miniaturized PCNL and flexible ureteroscopes. The novel super-mini-PCNL (SMP) device involves only a small percutaneous access, leading to small blood loss while remaining effective; in addition, the visual field is appropriate, the procedure is short, and the device is easy to operate [9, 10]. SMP is considered feasible and safe for moderate calculi in adults and children, but the use of SMP in patients with larger calculi remains uncertain.

Therefore, this meta-analysis aimed to review the efficacy (stone-free rate, SFR) and safety of SMP in treating urinary calculi. The results could help strengthen the indications of SMP and improve patient management.

## Methods

### Evidence acquisition

#### Literature search

The present systematic review and meta-analysis was performed according to the Preferred Reporting Items for Systematic Reviews and Meta-Analyses (PRISMA) guidelines [11]. The meta-analysis was designed using the PICOS principle [12]. PubMed, Embase, and the Cochrane Library were searched for potentially eligible studies published up to May 2021, followed by screening based on the inclusion and exclusion criteria. The search terms were ‘Super-mini PCNL’ and ‘urinary calculi’. The literature search and study identification process were performed independently and in parallel by two investigators (Han Li andYong Yin). Discrepancies in study selection were resolved by discussion until consensus. The reference lists of the identified reports were screened for additional studies that might qualify. For papers reporting the same study population, only the one with the highest quality assessment was included.

#### Eligibility criteria

The inclusion criteria were (1) patients: adults diagnosed with nephrolithiasis, (2) intervention: SMP, (3) comparison: not limited, (4) primary outcome: SFR, (5) secondary outcome: complications (using the Clavien-Dindo grading system [13]), pain score, hospitalization days, and mean hemoglobin decline, (6) language: English, and (7) the full text was available. The exclusion criteria were (1) non-human study, (2) case report, (3) case series, or (4) review, meta-analysis, or comments.

#### Data extraction

The study characteristics (first author, publication years, study design, country, and control group), patient’s characteristics (sex, sample size, age, calculi size, calculi side, and calculi site), and outcomes (SFR, complications, hospitalization days, mean hemoglobin decline, and pain scores) were extracted and reviewed by two investigators (Han Li andYong Yin). Discrepancies were solved by discussion until a consensus was reached.

#### Quality of the evidence

The level of evidence of the articles was assessed independently by two authors (Han Li andYong Yin) according to Version 2 of the Cochrane risk-of-bias assessment tool for randomized controlled trials (Rob 2) (RCTs) [14] and the Newcastle-Ottawa Scale (NOS) criteria for cohort studies [15]. Discrepancies in the assessment were resolved by discussion.

### Statistical analysis

All analyses were performed using STATA SE 14.0 (StataCorp, College Station, TX, USA). Statistical heterogeneity among studies was calculated using Cochran’s Q-test and the I^2^ index. An I^2^ > 50% and Q-test P < 0.10 indicated high heterogeneity. The meta-analysis was performed using a random-effects model to avoid overestimation. P < 0.05 were considered statistically significant. The SFR and complications were summarized as risk ratios (RRs) with 95% confidence intervals (CIs). The standardized mean differences (SMDs) and 95% CIs were used for the mean hemoglobin decline, whereas weighted mean differences (WMDs) with their 95% CIs were used for the other continuous variables. The potential publication bias was not assessed using funnel plots and Egger’s test because the numbers of studies included in each quantitative analysis were less than 10, in which case the funnel plots and Egger’s test can yield misleading results [16].

## Results

### Study selection

**Fig. 1:**
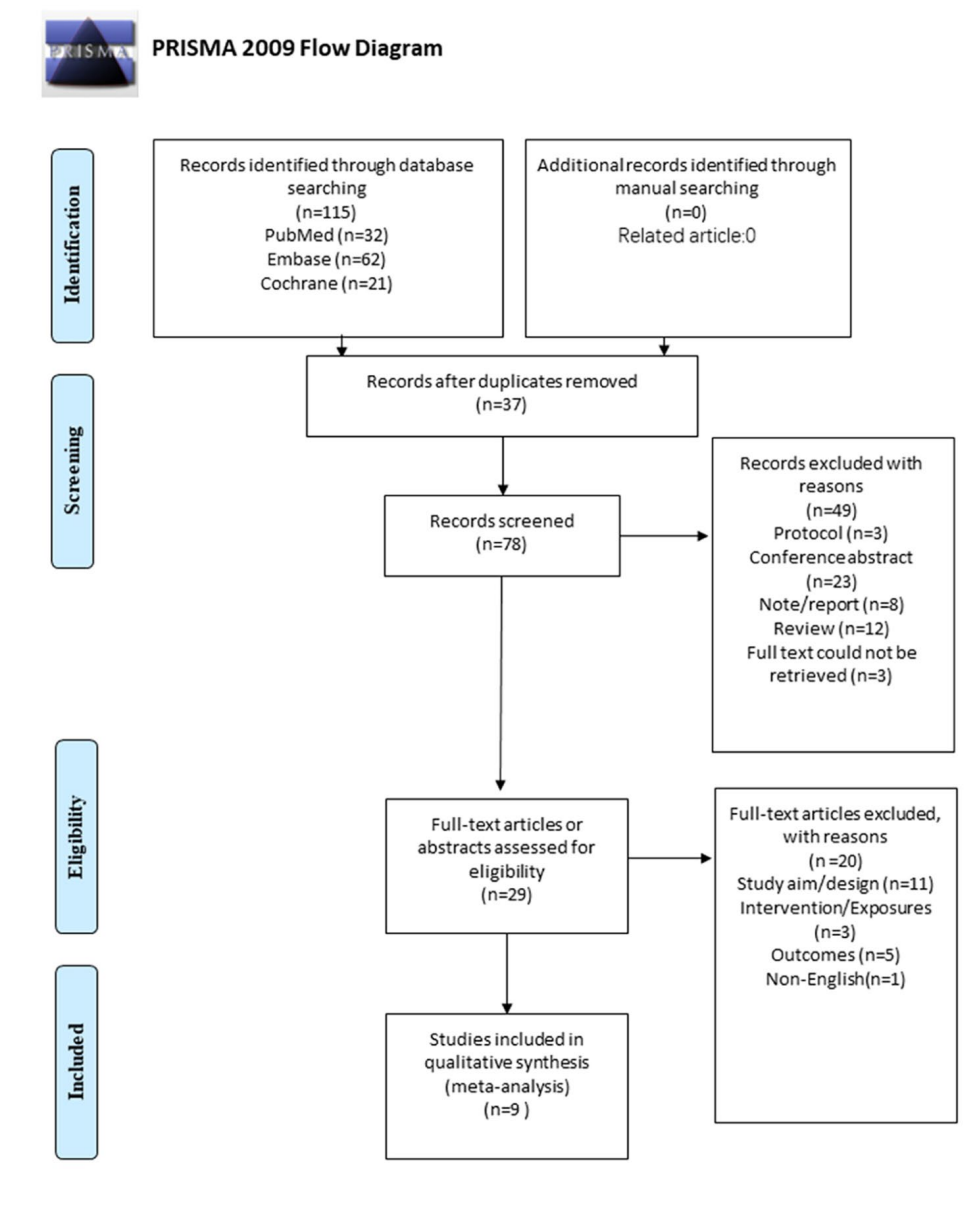
PRISMA 2009 Flow Diagram

Figure 1 presents the study selection process. The initial search yielded 115 records; 37 were duplicates and were removed. Then, 78 records were screened, and 49 were excluded. Twenty-nine full-text articles or abstracts were assessed for eligibility, and 20 were excluded (study aim/design, n = 11; intervention/exposures, n = 3; outcomes, n = 5; non-English, n = 1). Finally, nine reports were included in this meta-analysis.

### Characteristics of the studies

Table 1 presents the characteristics of the included studies. There were three RCTs [17–19], one prospective cohort study [20], and five retrospective cohort studies [21–25]. One study was from India and Turkey [17], one was from China and India [18], and seven were from China [19, 20, 22–25]. A total of 2433 patients underwent SMP, and 2178 patients underwent the control procedure. The mean age was in the late forties in seven studies [17–21, 23, 24], and two studies involved children [22, 25].

**Table 1:** Characteristics of the included studies

Author, year	Study design	Country	Control group	Sample size		Age, years		Sex, % male		Stone size, cm		Stone side		Stone site		Population
				Intervention	Control	Intervention	Control	Intervention	Control	Intervention	Control	Left	Right	Single	Multiple	
Guddeti 2020 ^27^	RCT	India, Turkey	sPNL	75	75	48.36 (19–76)	46.53 (20–80)	76	68	1.48 (0.78)	1.49 (0.73)	37/38	32/43	/	/	< 2 cm
Zeng 2018 ^28^	RCT	China, India	RIRS	80	80	49.4 (12.8)	47.1 (13.9)	62.5	57.5	1.5 (0.29)	1.43 (0.34)	38/42	42/38	/	/	1–2 cm
Zhong 2020 ^29^	RCT	China	mini-PCNL	46	47	49.1 (9.3)	50.5 (10.5)	60.8	66	3.27 (0.85)	3.28 (0.93)	/	/	/	/	2–5 cm
Liu 2018 ^30^	Prospective cohort	China	Miniperc	79	257	45.8 (14.4)	18.7 (11.3)	64.6	58.4	3.0 (1.0)	3.0 (0.8)	/	/	28/54	37/159	> 2 cm
Gao 2019 ^31^	Retrospective cohort	China	f-URS	40	55	52.88 (13.08)	49.04 (14.25)	57.5	65.5	2.4 (0.8)	2.3 (1.1)	21/24	19/31	/	/	/
Jia 2020 ^32^	Retrospective cohort	China	RIRS	36	25	4.5 (2.7)	4.3 (2.5)	72.2	60	1.42 (0.3)	1.4 (0.28)	14/10	22/15	26/18	10/7	1–2 cm
Liu 2020 ^33^	Retrospective cohort	China	Miniperc	2012	1513	46.5 (16)	50.8 (11.5)	67.1	55.6	3.11 (0.9)	3.2 (0.88)	/	/	557/428	1455/1085	> 2 cm
Xu 2020 ^34^	Retrospective cohort	China	f-URS	48	104	49.96 (12.86)	48.72 (13.56)	70.8	68.3	/	/	27/57	21/47	19/54	29/50	2–3 cm
Yuan 2019 ^35^	Retrospective cohort	China	mini-PCNL	17	22	7.8 (3.5)	9.2 (3.8)	64.7	68.2	/	/	7/9	6/13	/	/	/

Abbreviation: RIRS, Retrograde intrarenal surgery; f-URS, flexible ureterorenoscopy; sPNL, standard percutaneous nephrolithotomy

Two RCTs [17, 18] had a high risk of bias for two items of the Rob 2 tool and an unclear risk of bias for one item, and one RCT [19] had an unclear risk of bias for three items (Additional File Table S1). Among the cohort studies, one study scored four stars on the NOS [24], one scored 6 stars [22], three scored 7 stars [21, 23, 25], and one scored 8 stars [20] (Additional File Table S2).

### Stone-free rate

**Fig. 2:**
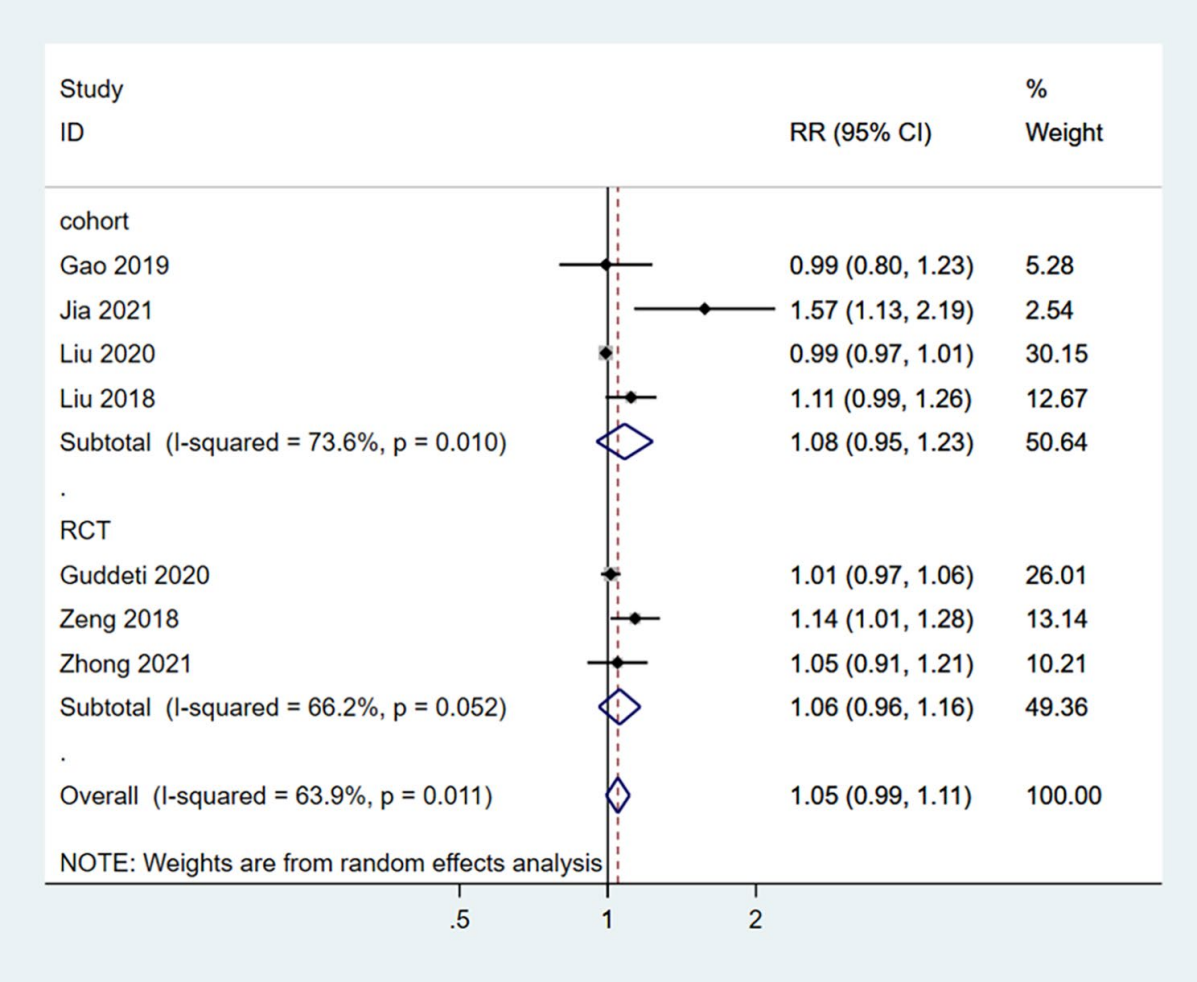
Forrest plot of the stone-free rate

Seven studies reported the SFR [17–23]. SMP was not associated with an improved SFR in patients with calculi (RR = 1.05, 95%CI: 0.99–1.11; I^2^ = 63.9%, P_heterogeneity_=0.011) (Fig. 2). Similar results were observed when considering the RCTs and cohort studies separately.

### Complications

**Fig. 3:**
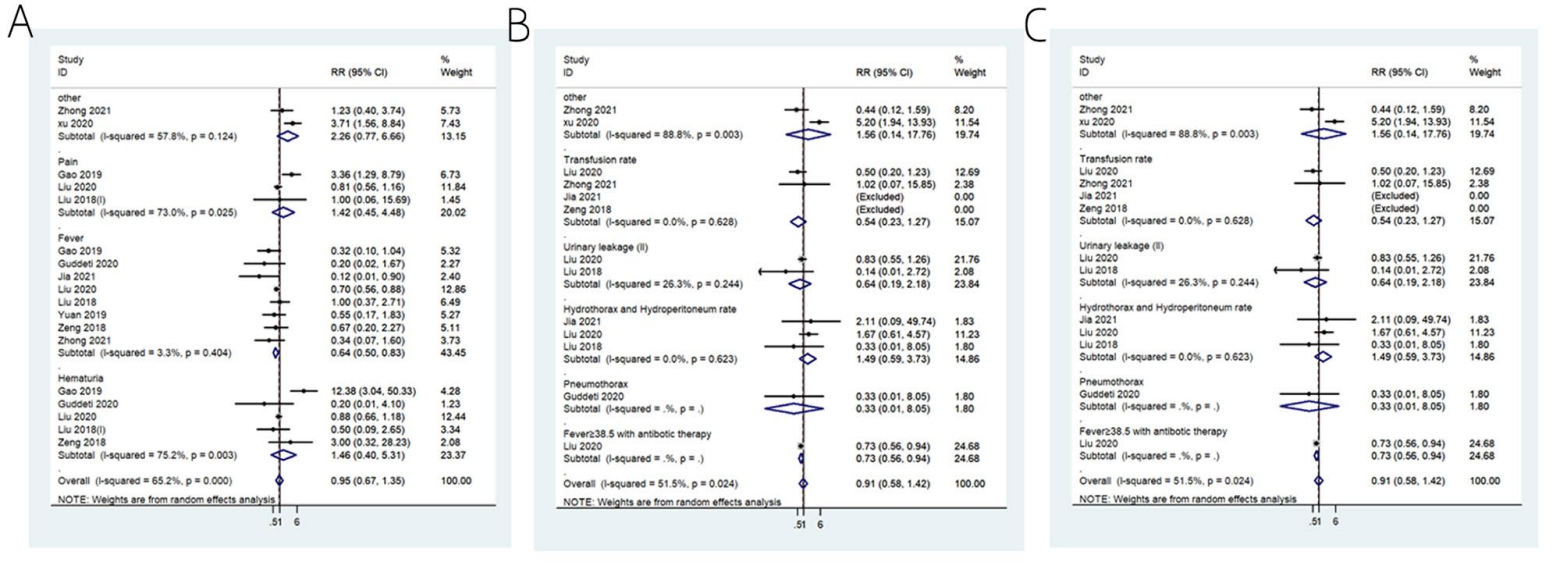
** A**: Forrest plot of the Clavien-Dindo I complications. **B**: Forrest plot of the Clavien-Dindo II complications. **C**: Forrest plot of the Clavien-Dindo III complications

All nine studies reported various complications [17–25]. There were no differences in the occurrence of Clavien-Dindo I (RR = 0.95, 95%CI: 0.67–1.35; I^2^ = 65.2%, P_heterogeneity_<0.001) (Fig. 3A) and Clavien-Dindo II (RR = 0.91, 95%CI: 0.58–1.42; I^2^ = 51.5%, P_heterogeneity_=0.024) (Fig. 3B) complications between SMP and the control procedures. There were more Clavien-Dindo III complications with SMP than with the control procedures (RR = 0.71, 95%CI: 0.55–0.91; I^2^ = 0.0%, P_heterogeneity_=0.541), but none of the individual complications were significantly different between the two groups (Fig. 3C). Clavien-Dindo I fever appeared to be higher with SMP than with the control procedures (RR = 0.64, 95%CI: 0.50–0.83; I^2^ = 3.3%, P_heterogeneity_=0.404) (Fig. 3A).

### Pain

**Fig. 4:**
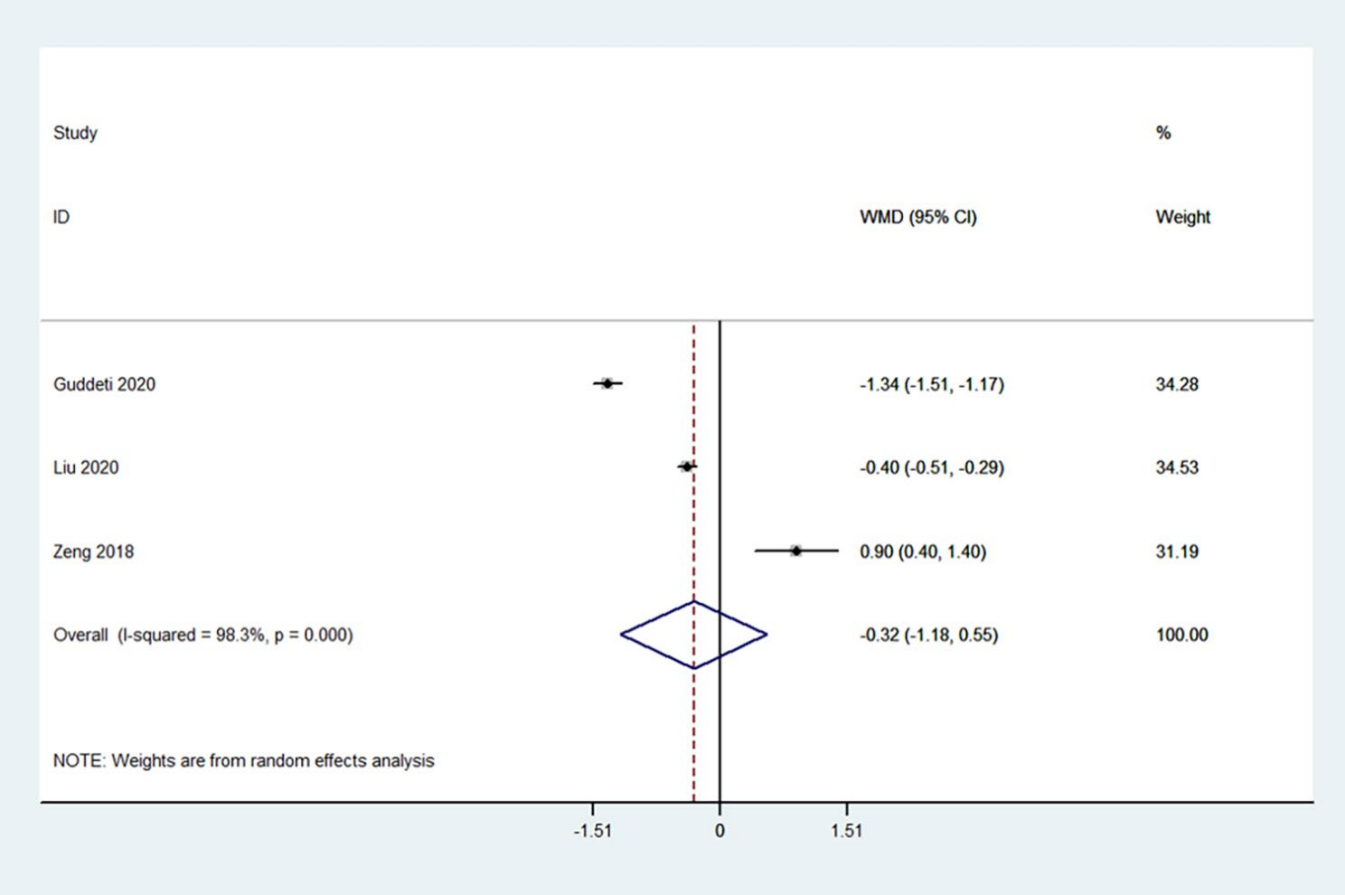
Forrest plot of the pain scores

Three studies presented pain data [17, 18, 23]. The pain was not significantly different between SMP and the control procedures (WMD=-0.32, 95%CI: -1.18-0.55; I^2^ = 98.3%, P_heterogeneity_<0.001) (Fig. 4).

### Length of hospitalization

**Fig. 5:**
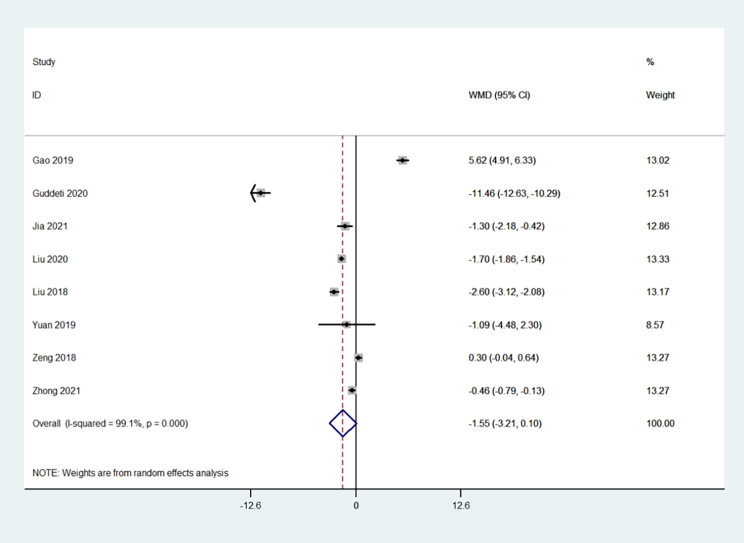
Forrest plot of the hospitalization period (days)

Eight studies presented hospitalization data [17–25]. The procedure was not associated with the length of hospital stay (WMD=-1.55, 95%CI: -3.21-0.10; I^2^ = 99.1%, P_heterogeneity_<0.001) (Fig. 5).

### Procedure duration

Eight studies presented surgery data [17–25]. SMP was not associated with the duration of surgery (WMD=-0.96, 95%CI: -7.34-9.25; I^2^ = 95.9%, P_heterogeneity_<0.001) (Additional File Figure S1).

## Discussion

The use of PCNL for calculi clearance is supported by guidelines and has good efficacy [1, 3–6] but carries a high risk of hemorrhage [7], complications, longer hospital stays, and even death [26]. Therefore, the SMP was designed to maintain de efficacy of PCNL while reducing the risk of hemorrhage [8]. The SMP is safe and feasible in adults and children with moderate-size renal calculi [9, 10]. Hence, due to the lower risk of complication and shorter hospitalization, miniature PCNL has become popular because of its better cost-effectiveness than other procedures, as suggested by recent reviews [27, 28].

Nevertheless, the effectiveness and safety of SMP for larger calculus burdens remain to be determined. Therefore, this study aimed to review the efficacy and safety of SMP in treating urinary calculi. The results suggested no difference in efficacy between SMP and other procedures in the treatment of urinary calculi of moderate size. Clavien-Dindo I fever and Clavien-Dindo III complications, in general, might be more frequent with SMP than with other procedures.

Thapa et al. [29] performed a systematic review (but not a formal meta-analysis) of 19 reports of mini-PCNL vs. standard PCNL. They showed that mini-PCNL improved the complication rates and length of hospitalization, but they did not include SMP. Of note, SMP is different from mini-PCNL. Indeed, SMP was designed by Zeng et al. [30] and consisted of a modified 10–14 F access sheath with suction and evacuation functions and a 7-F nephroscope with an enhanced irrigation feature. SMP aims to efficiently remove calculus fragments at low intrapelvic pressure [30]. In first-generation SMP, the sheath was made of clear plastic and could bend easily [30]. First-generation SMP effectively dealt with calculi of < 25 mm but led to more complications and longer hospitalization for large calculi [30, 31]. Then, in second-generation SMP, the sheath is made of metal and has a higher irrigation-suction efficiency than the first-generation system [9, 30]. A study showed that the second-generation SMP could deal with calculi of > 20 mm with low complication rates [31].

The present meta-analysis showed no differences in SFR between SMP and the control procedures. SMP showed higher frequencies of Clavien-Dindo I fever and Clavien-Dindo III complications, but no individual Clavien-Dindo III complication seems to drive the difference. Of note, heterogeneity was high for most analyses. Indeed, the included studies differed in sample size, populations, control procedure, and first/second-generation SMP. The patients with larger calculi operated with first-generation SMP might drive the differences in complications, as observed in the first studies of SMP [30, 31]. Indeed, all included studies examined calculi of ≥ 10 mm. Therefore, these results should be taken cautiously, pending well-designed studies that compare SMB with other procedures. A prospective cohort study aiming at 3000 participants is currently underway to compare standard PCNL, mini-PCNL, and second-generation SMP (ClinicalTrials.gov NCT03771365).

This meta-analysis has limitations. First, all meta-analyses inherit the limitations of all included studies, and caution must be applied while extrapolating the results. Second, different surgeons might have had different experiences with SMP and other interventions, affecting the outcomes. In addition, we did not severely limit the types of studies that could be included. Therefore, a further meta-analysis that would include only prospective, randomized, and multicenter RCTs would be necessary for a more comprehensive and convincing evaluation in the future. Third, most included studies were performed in China. It is probably because SMP was first created and implemented in China [9, 10, 31]. If additional countries conduct such studies in the future, we will update the meta-analysis to increase the credibility of the results.

## Conclusion

In conclusion, the results suggested no difference in efficacy between SMP and other procedures in the treatment of urinary calculi of moderate size. Clavien-Dindo I fever and Clavien-Dindo III complications, in general, might be more frequent with SMP than with other procedures.

## Electronic supplementary material

Below is the link to the electronic supplementary material.

Supplementary Table S1: ROB 2.0 for quality assessment of RCTs

Supplementary Figure S1: Forrest plot of the length of operation (minutes)

## Data Availability

All data generated or analyzed during this study are included in this published article.
