# Supplementary material for: Efficacy and safety of super-mini percutaneous nephrolithotomy in the treatment of urinary calculi: a systematic review and meta-analysis
Source: BMC Urol. 2023 May 9;23:87. doi: 10.1186/s12894-023-01256-z (PMC10170803; doi:10.1186/s12894-023-01256-z)
Supplement: Supplementary file 1 — Supplementary Table S1: ROB 2.0 for quality assessment of RCTs [file 12894_2023_1256_MOESM1_ESM.docx]

**Supplementary Table S1.** ROB 2.0 for quality assessment of RCTs

| Study | Bias arising from the randomization process | Bias due to deviations from intended interventions | Bias due to missing  outcome data | Bias in measurement of the outcome | Bias in selection of the  reported result | Overall bias |
| --- | --- | --- | --- | --- | --- | --- |
| Guddeti 2020 [27] | Unclear | High | Low | Low | Low | High |
| Zeng 2018 [28] | Unclear | High | Low | Low | Low | High |
| Zhong 2020 [29] | Unclear | Unclear | Low | Low | Low | Unclear |

**Supplementary Table S2.** NOS criteria for the cohort studies

| Study | Representativeness of the exposed cohort | Selection of the non-exposed cohort | Ascertainment of exposure | Demonstration that outcome of interest was not present at the start of the study | Comparability of cohorts based on the design or analysis | Assessment of outcome | Was follow-up long enough for outcomes to occur | Adequacy of follow up of cohorts | Total quality scores |
| --- | --- | --- | --- | --- | --- | --- | --- | --- | --- |
| Liu 2018 [30] | ⭐ | ⭐ | ⭐ | ⭐ | ⭐⭐ | / | ⭐ | ⭐ | 8 |
| Gao 2019 [31] | ⭐ | ⭐ | ⭐ | / | ⭐ | ⭐ | ⭐ | ⭐ | 7 |
| Jia 2020 [32] | ⭐ | ⭐ | ⭐ | / | ⭐ | / | ⭐ | ⭐ | 6 |
| Liu 2020 [33] | ⭐ | ⭐ | ⭐ | / | ⭐⭐ | / | ⭐ | ⭐ | 7 |
| Xu 2020 [34] | / | / | ⭐ | / | ⭐ | / | ⭐ | ⭐ | 4 |
| Yuan 2019 [35] | ⭐ | ⭐ | ⭐ | / | ⭐⭐ | / | ⭐ | ⭐ | 7 |
