# Supplementary figures and images for: Efficacy and safety of super-mini percutaneous nephrolithotomy in the treatment of urinary calculi: a systematic review and meta-analysis
Source: BMC Urol. 2023 May 9;23:87. doi: 10.1186/s12894-023-01256-z (PMC10170803; doi:10.1186/s12894-023-01256-z)

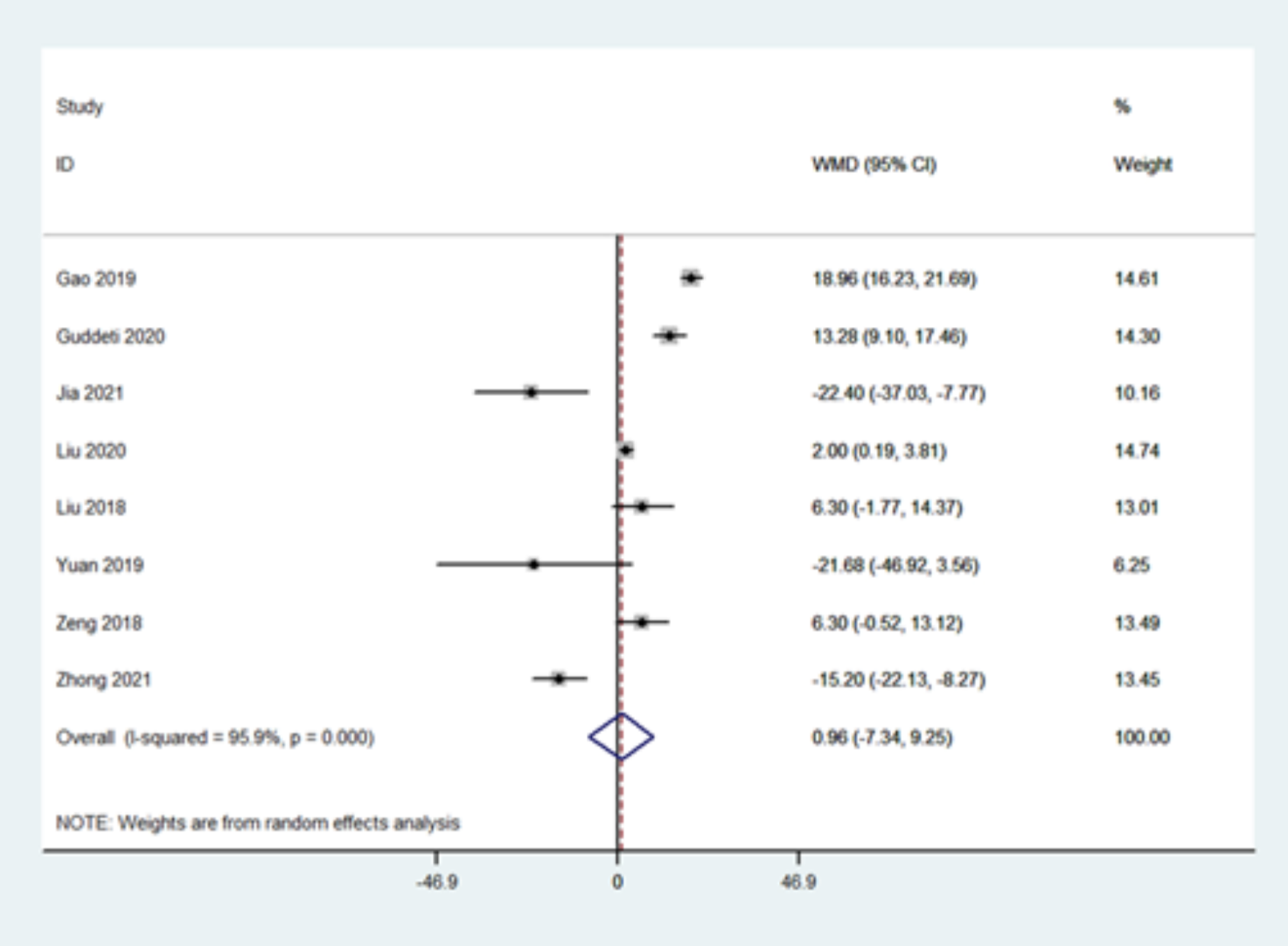


**Supplementary Figure S1.** Forrest plot of the length of operation (minutes)

Supplement: Supplementary file 2 — Supplementary Figure S1: Forrest plot of the length of operation (minutes) [file 12894_2023_1256_MOESM2_ESM.docx]
